# Non-invasive Investigations for the Diagnosis of Fontan-Associated Liver Disease in Pediatric and Adult Fontan Patients

**DOI:** 10.3389/fcvm.2017.00015

**Published:** 2017-03-27

**Authors:** Amyna Fidai, Frederic Dallaire, Nanette Alvarez, Yvonne Balon, Robin Clegg, Michael Connelly, Frank Dicke, Deborah Fruitman, Joyce Harder, Kimberley Myers, David J. Patton, Tim Prieur, Erika Vorhies, Robert P. Myers, Steven R. Martin, Steven C. Greenway

**Affiliations:** ^1^Department of Paediatrics, Cumming School of Medicine, Alberta Children’s Hospital Research Institute, University of Calgary, Calgary, AB, Canada; ^2^Division of Pediatric Cardiology, University of Sherbrooke, Centre de Recherche du Centre Hospitalier Universitaire de Sherbrooke, Sherbrooke, QC, Canada; ^3^Libin Cardiovascular Institute of Alberta, University of Calgary, Calgary, AB, Canada; ^4^Liver Unit, Division of Gastroenterology, Cumming School of Medicine, University of Calgary, Calgary, AB, Canada

**Keywords:** liver, Fontan, Fontan-associated liver disease, congenital heart disease, fibrosis

## Abstract

Fontan-associated liver disease (FALD) is a serious complication related to the chronically elevated venous pressure and low cardiac output of this abnormal circulation. However, diagnostic markers for this condition are limited. We hypothesized that specific tests for fibrosis developed for other chronic liver diseases would identify a higher prevalence of FALD than ultrasound and standard laboratory tests and that identified abnormalities would correlate with time post-Fontan. In this cross-sectional study, we assessed 19 children (average age 8.4 ± 4.3 and 5.4 ± 4.1 years post-Fontan) and 8 adults (average age 31.5 ± 8.9 and 21.1 ± 4 years post-Fontan) using standard serum laboratory investigations assessing hepatic integrity and function, the FibroTest, liver ultrasound, and transient elastography (FibroScan). In adult Fontan patients, hemoglobin, C-reactive protein, and gamma-glutamyl transpeptidase were significantly increased, and white blood cell and platelet counts were significantly decreased in comparison to the pediatric cohort. International normalized ratio was mildly elevated in both children and adults. FibroTest results were suggestive of fibrosis regardless of time post-Fontan. FibroScan measurements were significantly correlated with time post-Fontan, but the incidence of ultrasound-detected liver abnormalities was variable. No cases of hepatocellular carcinoma were identified. Abnormalities suggestive of FALD occur in both children and adults post-Fontan. Select laboratory tests, and possibly ultrasound and FibroScan in some patients, appear to have the most promise for the non-invasive detection of FALD.

## Introduction

Currently performed as a series of palliative surgeries, the Fontan circulation separates venous return from the heart allowing volume unloading of the single ventricle and permitting normal arterial oxygen saturations. However, this is achieved at the expense of elevated central venous pressure and decreased cardiac output. Intolerance of the surgically created Fontan physiology can arise early or, more commonly, decades later during adulthood (1).

Liver disease, which includes cirrhosis, ascites, synthetic dysfunction, hepatocellular carcinoma (HCC), and portal hypertension, is increasingly recognized as a potentially serious morbidity post-Fontan (2–4). Fontan-associated liver disease (FALD) is defined as abnormalities in liver structure and function resulting from the Fontan circulation and not related to another process (e.g., viral hepatitis, medications, or alcohol toxicity) (5). However, the prevalence of FALD is not well defined. Liver histology post-Fontan has been reviewed in multiple small case series including autopsy reviews and liver biopsies, and, in general, more severe disease is identified in patients further out from Fontan completion (6–8).

The gold standard for the assessment of liver fibrosis is a liver biopsy, but this invasive procedure is not widely used for routine patient surveillance and requires an anesthetic in young patients, which may result in significant complications, and its accuracy for the detection of disease is limited to the area sampled. Some effective non-invasive methods of detection that have been investigated include looking for peripheral biomarkers of fibrosis in the blood using the FibroTest algorithm (9) and assessing liver elasticity, including transient elastography or FibroScan (10, 11). These tests for liver fibrosis have been well validated in other chronic liver diseases (e.g., viral hepatitis) but are relatively understudied post-Fontan (12, 13).

The goals of this study were to assess for signs of FALD in a group of pediatric and adult Fontan patients using non-invasive standard investigations as well as the FibroTest and FibroScan and identify potentially useful diagnostic tools.

## Materials and Methods

### Study Population

This cross-sectional study was approved by the Conjoint Health Research Ethics Board at the University of Calgary (ID REB13-0663) and carried out between September 2013 and April 2014. Patients eligible for inclusion were all stable patients post-Fontan within our catchment area of southern Alberta. Subjects were excluded if there was a history of non-cardiac liver disease (i.e., viral hepatitis, excessive alcohol consumption) or other complications related to the Fontan physiology (e.g., protein-losing enteropathy). Informed written consent was obtained from all participants or their parent in accordance with the Declaration of Helsinki. Patient demographics and prior surgical details were obtained from the medical chart.

### Investigations

Multiple laboratory investigations were measured using patient serum. Tests performed included complete blood count (CBC), C-reactive protein (CRP), and α-fetoprotein (AFP). Liver synthetic and metabolic function was evaluated using markers of coagulation [international normalized ratio (INR), partial thromboplastin time (PTT), and factor VII] as well as albumin, total protein, and bilirubins (total and direct). Measured liver enzymes included alkaline phosphatase (ALP), alanine transaminase (ALT), aspartate transaminase (AST), and gamma-glutamyl transpeptidase (GGT). The AST to platelet ratio index (APRI) was calculated as the ratio of the normalized AST and platelet count (14). The FibroTest (FibroSURE in the USA) was calculated from five serum markers including total bilirubin, GGT, haptoglobin, apolipoprotein-A1, and α-2-macroglobulin (9, 15).

An ultrasound of the liver (US) was requested to assess the liver parenchyma and identify any signs of portal hypertension. To enhance patient compliance and to reflect real-world testing, performance and reporting of the US were not standardized but all were performed in a clinically certified facility in Alberta, Canada. Each US was scored for the presence of (1) abnormal liver parenchyma, (2) presence of hepatic nodules, (3) evidence of portal hypertension (i.e., reversal of portal venous flow, omental thickening, varices), and (4) the presence of splenomegaly. The relative number of abnormalities reported was used to calculate an US-based score for each patient with 0 indicating no abnormalities and 1 indicating all 4/4 abnormalities present.

Transient elastography (FibroScan, Echosens, France) using the M probe was used for all Fontan patients and was performed by a single experienced operator in a standardized manner reporting the median value of at least 10 measurements from the right lobe of the liver (16). All scans were interpreted by an expert hepatologist (RPM), and liver stiffness was reported in kilopascals.

### Statistics and Data Analysis

Laboratory data were compared to age-appropriate normal values provided by the performing clinical laboratory (Calgary Laboratory Services, Calgary, AB, Canada). Prevalence of abnormal test results is expressed as the percentage of subjects above the upper limit of normal for each test. Most variables were not normally distributed, and results are expressed as median and ranges (10th and 90th percentiles). Medians between groups were compared using a non-parametric Wilcoxon two-sample test. Linear correlations between liver function tests and time post-Fontan were assessed using Spearman rank correlation coefficient. The presence of a significant difference between the number of abnormalities in each group (children vs. adults) was determined using Fisher exact probability test. A *p* < 0.05 was considered statistically significant. All analyses were performed using SAS for Windows version 9.4 (SAS Institute Inc., Cary, NC, USA). Graphs and linear regression were generated using Prism 7 for Mac OS X (GraphPad Software, La Jolla, CA, USA).

## Results

### Patient Characteristics

Our two patient groups (Table 1) included 19 children and 8 adult Fontan patients. There were clear and expected differences between the two groups with regards to age and time post-Fontan, and the adult group had, on average, undergone Fontan completion later than the pediatric group reflecting prior surgical practice. In the group of pediatric subjects, the dominant ventricle was primarily a morphological right ventricle in contrast to the adult group. Most children had undergone an extracardiac Fontan, whereas the adult group was more evenly distributed.

**Table 1:** **Study patient characteristics**.

Variable	Children	Adults
Number of patients	19	8
Age (years)	7.0 (3, 16.8)	29.0 (21–44)
Age at Fontan (years)	3.1 (1.9, 3.8)	6.4 (2.3–28.1)
Time post-Fontan (years)	3.7 (1.1, 14.4)	20.6 (15.9–29.2)
Male (%)	12 (63)	6 (75)
Functionally univentricular RV	14	0
Functionally univentricular LV	5	8
Extracardiac Fontan	13	3
Lateral tunnel Fontan	3	3
Unknown type of Fontan	3	2

*Results are shown as the median (10th, 90th percentiles) or *n* number of patients*.

### Laboratory Investigations

Multiple differences in routine serum laboratory tests were noted between the pediatric and adult Fontan groups (Table 2). In comparison to the pediatric patients, white blood cell (WBC) and platelet counts were significantly decreased in the adult Fontan patients, and hemoglobin was significantly increased. Systemic inflammation was investigated using CRP and the median value was normal in both adults and children. AFP was not elevated beyond the reference range in any patient. A mild coagulopathy was noted in all patients with the median INR in both groups being above the normal reference range. PTT and factor VII were also noted to be at the upper range of normal for all patients.

**Table 2:** **Results of serum laboratory investigations for the pediatric and adult groups**.

Test	Children			Adults			*p*
	Results	Abnormal results (%)	Reference range	Results	Abnormal results (%)	Reference range	
**WBC**	**7.1 (4.7, 11.1)**	**0**	**4–14**	**4.4 (4.1, 8.1)**	**13**	**4–11**	**0.038**
**Hemoglobin**	**145 (133, 165)**	**7**	**110–157**	**171 (171, 177)**	**25**	**137–180**	**0.0001**
**Platelets**	**232 (129, 323)**	**14**	**150–400**	**120 (109, 183)**	**75**	**150–400**	**0.0004**
INR	1.3 (1.1, 1.6)	86	0.9–1.1	1.2 (1.1, 1.6)	50	0.9–1.1	0.38
PTT	34 (31, 40)	43	25–35	34 (33, 38)	25	25–35	0.36
**CRP**	**0 (0, 2.6)**	**0**	**0–8**	**2.2 (1.2, 8.8)**	**13**	**0–8**	**0.0004**
**AFP**	**1.2 (0.5, 3.6)**	**0**	**0–10**	**2.3 (1.9, 3.5)**	**0**	**0–10**	**0.0044**
Albumin	41 (37, 44)	0	33–48	43 (43, 47)	0	33–48	0.074
**Total protein**	**66 (63, 75)**	**0**	**63–80**	**76 (69, 83)**	**25**	**63–80**	**0.015**
**ALP**	**225 (120, 382)**	**0**	**45–450**	**97 (35, 147)**	**38**	**30–130**	**0.0002**
ALT	19 (13, 30)	0	1–35	28 (16, 44)	13	1–60	0.16
AST	30 (24, 37)	0	10–45	27 (22, 34)	13	8–40	0.19
α-2-Macroglobulin	3.2 (2, 3.6)	71	1.02–2.59	2.5 (2.8, 3.3)	38	1.02–2.59	0.15
**Bilirubin (total)**	**8 (4, 21)**	**21**	**0–14**	**14 (14, 27)**	**43**	**0–24**	**0.027**
**Bilirubin (direct)**	**3 (1, 6)**	**0**	**0–7**	**5.5 (4, 8)**	50*	**0–7**	**0.032**
**GGT**	**42 (24, 89)**	**29**	**11–63**	**67 (43, 332)**	**63**	**11–63**	**0.05**
Haptoglobin	0.3 (0.06, 1.2)	50	0.3–2	0.4 (0.3, 0.9)	38	0.3–2	0.75
Apolipoprotein-A1	1.17 (0.9, 1.6)	29	1.04–2.02	1.28 (1.24, 1.42)	0	1.04–2.02	0.18
Factor VII	0.54 (0.29, 0.74)	36	≥0.50	0.49 (0.62, 0.68)	67	≥0.50	0.77
FibroTest	0.45 (0.22, 0.69)	93	<0.21	0.61 (0.49, 0.75)	100	<0.21	0.13

*Results are shown as median (10th, 90th percentiles). Those tests that were significantly different between groups (*p* < 0.05) are highlighted in bold. For the pediatric group, *N* = 11–14 and for the adult group, *N* = 5–8*.

*WBC, white blood cell; INR, international normalized ratio; PTT, partial thromboplastin time; CRP, C-reactive protein; AFP, α-fetoprotein; ALP, alkaline phosphatase; ALT, alanine transaminase; AST, aspartate transaminase; GGT, gamma-glutamyl transpeptidase*.

*The number of abnormalities was significantly different between groups (*p* < 0.05).

**Figure 1:**
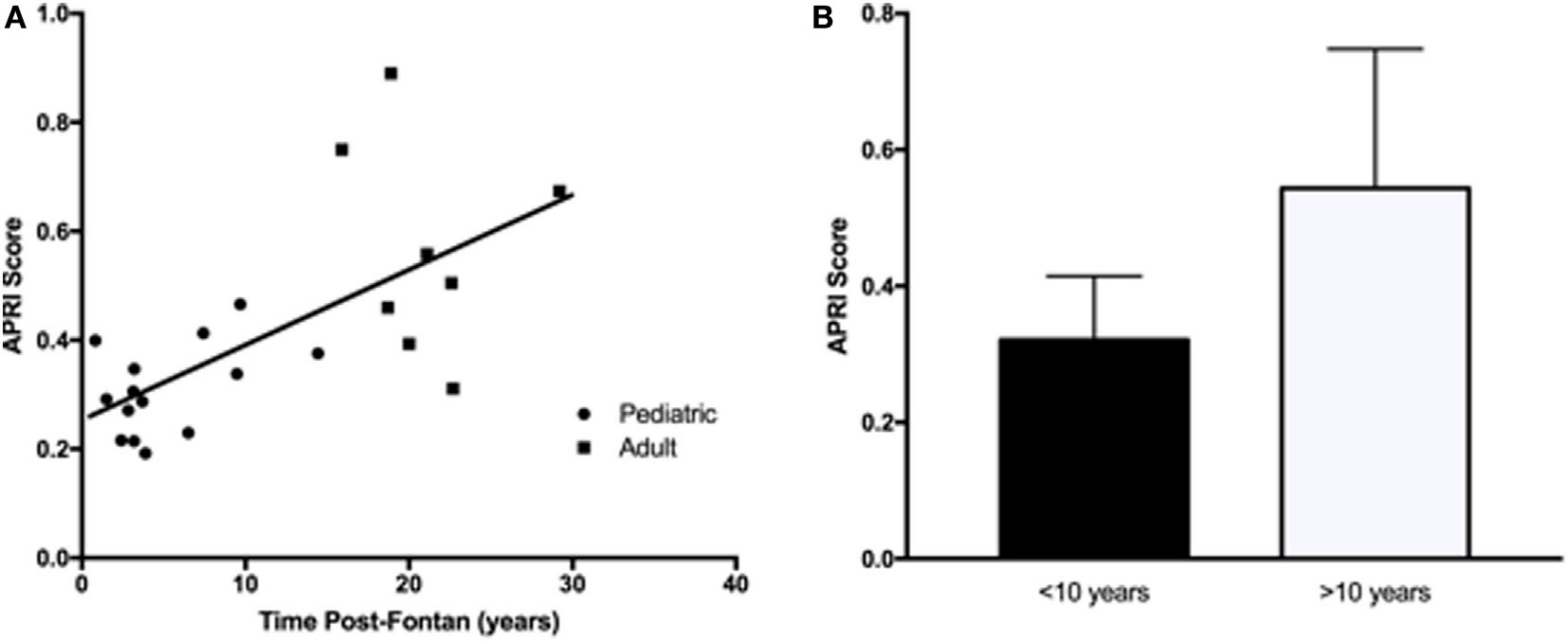
**The aspartate transaminase to platelet ratio index (APRI) score was elevated in adult Fontan patients and in those who were more than 10 years post-Fontan**. **(A)** There was a significant correlation (*R*^2^ = 0.36, *p* = 0.01) between an increasing APRI and time post-Fontan. **(B)** The APRI was significantly elevated (*p* = 0.02) in patients who were >10 years post-Fontan.

Liver protein synthetic function was within normal limits for all patients as indicated by the normal values for serum albumin and total protein. Serum bilirubin levels (total and direct) were significantly higher in the adult Fontan patients. ALT and AST were not significantly different between the groups and were not significantly increased above the reference range. However, GGT was increased in the adult patient group, with three patients demonstrating levels twofold to ninefold above the upper limit of normal. For GGT in the pediatric patients, the greatest abnormality was only 1.5-fold above the upper limit of normal. An elevated APRI was significantly correlated (*R*^2^ = 0.36, *p* = 0.01) with increasing time post-Fontan (Figure 1A), and when patients were dichotomized into those less than or more than 10 years post-Fontan, the APRI was significantly elevated (*p* = 0.02) in patients who were >10 years post-Fontan (Figure 1B).

**Figure 2:**
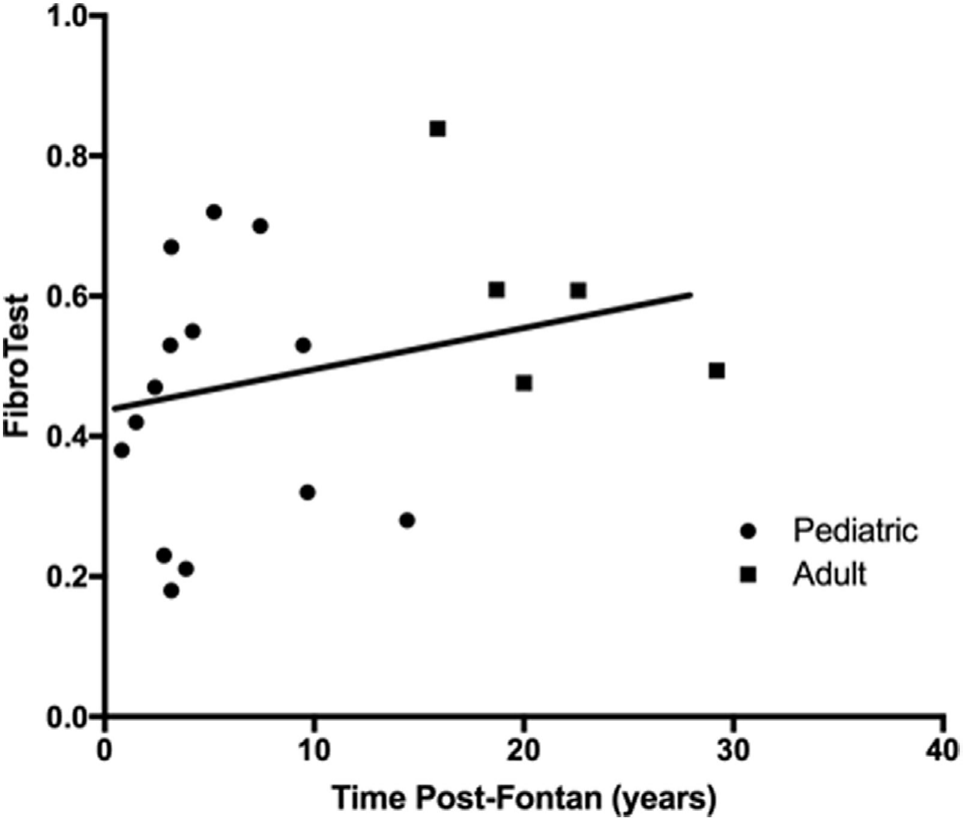
**The FibroTest was abnormal for all patients regardless of time post-Fontan**. No significant difference was observed between the pediatric and adult groups (*R*^2^ = 0.073, *p* = 0.26). A FibroTest score >0.48 indicates stage 2 fibrosis and >0.75 indicates cirrhosis.

Components of the FibroTest (haptoglobin, apolipoprotein-A1) were within normal limits, but the median α-2-macroglobulin was above normal in the pediatric group although within normal limits for the adult patients. The calculated FibroTest (scale ranges from 0 to 1.0 with higher values indicating more severe disease) was markedly abnormal for all patients regardless of time post-Fontan (Figure 2), and there was no significant difference observed in the scores between the adult and pediatric groups (*R*^2^ = 0.073, *p* = 0.26) or when patients were dichotomized into less than and greater than 10 years post-Fontan (data not shown).

### Ultrasound

**Figure 3:**
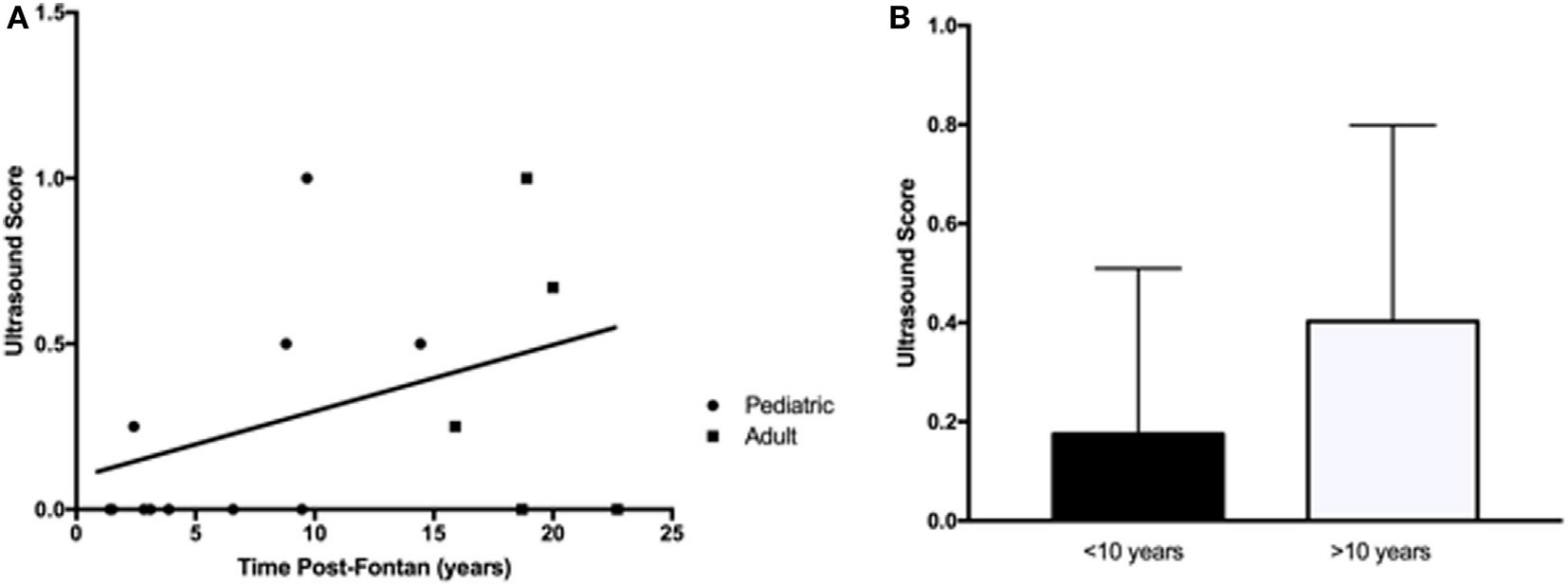
**Abnormalities detected by liver ultrasound did not increase with time post-Fontan**. **(A)** More adults (3/5) than children (4/10) demonstrated ultrasound abnormalities, but the increase over time was not statistically significant (*R*^2^ = 0.16, *p* = 0.12). **(B)** There was a trend toward more US abnormalities being present with increased time post-Fontan (>10 years), but this difference was not statistically significant (*p* = 0.27).

An abdominal US was requested to identify abnormal liver parenchyma, the presence of any intrahepatic nodules, assess for evidence of portal hypertension, and splenomegaly. There were more patients from the adult group with multiple US abnormalities (Table 3), but this increase over time (*R*^2^ = 0.16, *p* = 0.12) was not significant (Figure 3A). Similarly, when patients were dichotomized into those less than or more than 10 years post-Fontan, there was a trend toward more US abnormalities being present with increased time post-Fontan (Figure 3B), but this difference was also not statistically significant (*p* = 0.27).

**Table 3:** **Ultrasound results**.

Cohort	ID	Patient age (years)	Time post-Fontan (years)	Parenchyma	Nodules	PH	Splenomegaly	Total	Score
Child	1	6	3	0		0	0	0	0
Child	2	10	7				0	0	0
Child	3	6	2	0	0	0	1	1	0.25
Child	4	17	14	1			0	1	0.5
Child	5	7	4	0			0	0	0
Child	6	13	10	1			1	2	1
Child	7	13	9	0	0	0	0	0	0
Child	8	2	2				0	0	0
Child	10	12	9	1			0	1	0.5
Child	11	6	1	0		0	0	0	0
Child	12	6	3	0	0		0	0	0
Adult	2	41	20	1	1		0	2	0.67
Adult	3	33	19	1	1		1	3	1
Adult	4	44	16	0	1	0	0	1	0.25
Adult	6	25	23	0				0	0
Adult	7	21	19	0		0	0	0	0

*ID, patient identification; PH, portal hypertension*.

*0 indicates no abnormality was detected, blank indicates data were not reported, and 1 indicates the parameter was reported as abnormal. The total number of abnormalities was divided by the number of parameters evaluated to give the US score*.

### Transient Elastography (FibroScan)

**Figure 4:**
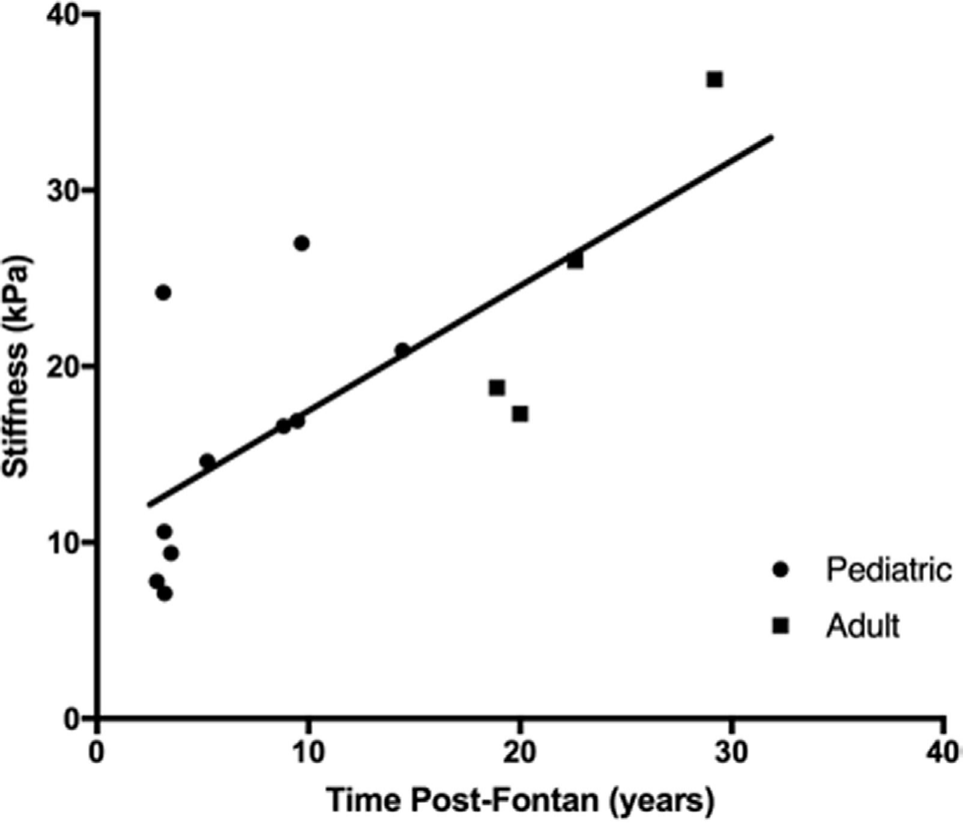
**Liver stiffness increased with time post-Fontan**. A progressive and significant increase in liver stiffness as measured using transient elastography (FibroScan) was noted with increasing time post-Fontan (*R*^2^ = 0.55, *p* = 0.0024).

The median liver stiffness score provided by the FibroScan was elevated for both the pediatric (14.6 kPa; 10th to 90th percentiles: 7.1–24.2 kPA) and adult (22.4 kPa, 10th to 90th percentiles: 17.3–36.3 kPa) Fontan patient groups and was borderline significantly different between the two groups (*p* = 0.056). However, there was a progressive and significant increase in liver stiffness with increasing time post-Fontan (Figure 4; *R*^2^ = 0.55, *p* = 0.0024).

## Discussion

We evaluated multiple investigations used in other chronic liver diseases for their applicability in detecting post-Fontan liver disease. Since FALD appears to be a progressive disease with increasing severity over time (17), we compared results from children to a group of adult Fontan patients as a surrogate for time post-Fontan. We also examined differences between groups of patients who were greater or less than 10 years post-Fontan to identify markers that potentially change over time and could therefore indicate FALD progression.

Liver-related abnormalities were observed in almost all participants, regardless of time post-Fontan completion. The INR was mildly abnormal in both patient groups, possibly related to subclinical liver disease or the consumption of clotting factors (18). Among the standard laboratory investigations, the WBC and platelet counts tend to decrease, while hemoglobin and GGT increase over time. The hematological alterations (increased hemoglobin, decreased platelets) likely reflect changes in the Fontan circulation. The increase in hemoglobin may reflect the development of venous collaterals and progressive relative cyanosis and the decrease in platelet count could be related to splenic sequestration due to progressive splenomegaly. This decrease in the platelet count is also responsible for the significant change in the APRI noted with increasing time post-Fontan. As has been documented previously, ALT and AST were generally unaffected post-Fontan with GGT being a more sensitive marker for liver injury (5, 19, 20). ALP was elevated in the pediatric cohort, but this reflects a developmental difference as indicated by the higher reference range for children. On the basis of US and serum AFP, we did not identify any cases of HCC in our patients, but the development of this disease remains a concern (21).

There was no significant difference in the FibroTest between the adults and children, and no significant increase was associated with increasing time post-Fontan. We hypothesize that the overall elevated scores of the FibroTest for all participants could be related to the presence of hepatic congestion or an alternative process (e.g., liver regeneration) created by the Fontan circulation, which could explain why this biomarker panel has not found utility in the detection of FALD (22).

Fontan-associated liver disease-related US findings in our patients were variable. The increase in US-detected abnormalities did not appear to increase significantly with increasing time post-Fontan. Use of a standardized protocol of reporting may be helpful in maintaining consistency of reporting. Our US results and the findings of others (23, 24) suggest that this test may be useful in following patients post-Fontan with the detection of portal hypertension or nodules, for example, being an indication for additional investigations and referral to a hepatologist.

Although for many patients the FibroScan was elevated and suggested the presence of fibrosis (and in some cases cirrhosis), there was a significant trend for increasing liver stiffness with increasing time post-Fontan. Although there is no way to non-invasively distinguish between hepatic congestion and fibrosis, this finding, supported by the observations of others (10, 25–27), suggests that transient elastography (or other measures of hepatic stiffness) could have utility in following at least some individual patients post-Fontan (i.e., those with a low stiffness early post-Fontan and acceptable pulmonary pressures) to monitor for worsening FALD. However, interpretation of the FibroScan should occur in the context of other liver- and cardiac-specific investigations, and the definitive diagnosis of fibrosis would still require a liver biopsy in these patients.

Our study has several important limitations. We made only indirect diagnoses of FALD, and we did not compare our results to liver biopsy. However, liver biopsy is not routinely used post-Fontan in most centers, and we sought to evaluate only non-invasive tests. Particularly for the FibroTest, the lack of biopsy data made interpretation difficult since it is known that hepatic congestion can cause abnormalities in these tests that purport to measure fibrosis (25) although it has recently been shown that no correlation exists between the FibroTest and biopsy score (22). Other limitations include the relatively small number of patients, but we did sample from the continuum of the Fontan age spectrum, which has not been commonly done in FALD studies. The incomplete collection of data for the US also limited its generalizability and strength of our conclusions.

## Conclusion

Fontan-associated liver disease is increasingly recognized as being widely prevalent post-Fontan and has potentially serious implications for patients. However, non-invasively distinguishing between hepatic congestion and fibrosis remains challenging. In a group of patients with a wide range of ages and time post-Fontan, we attempted to identify investigations that could aid in following patient liver health over time with the hypothesis that these fibrosis-specific tests would be useful in identifying FALD. Our study suggests that markers of liver fibrosis used in viral hepatitis (i.e., FibroTest) do not appear to provide reliable insight into progression of FALD-related fibrosis. For the surveillance of the liver in patients post-Fontan, we suggest serial monitoring of the CBC, INR, GGT, and AFP. Liver US allows for the identification of premalignant changes and signs of portal hypertension, which are clinically important. Serial transient elastography may have utility in some patients, but this remains to be defined in a longitudinal study. We conclude that no single test is predictive or even useful and results of liver testing should be interpreted in the context of hemodynamic information.

## Author Contributions

AF, SM, and SG designed the study. AF and SG performed the study. FD performed the statistical analyses. NA, YB, RC, MC, FD, DF, JH, KM, DP, TP, and EV assisted with patient recruitment and data collection. RM provided the FibroScan instrument and performed the FibroScan interpretation. AF and SG wrote the manuscript. All authors reviewed the manuscript.

## Conflict of Interest Statement

The authors declare that the research was conducted in the absence of any commercial or financial relationships that could be construed as a potential conflict of interest. The reviewer EA and handling Editor declared their shared affiliation, and the handling Editor states that the process nevertheless met the standards of a fair and objective review.

## Abbreviations

AFP, α-Fetoprotein; ALP, alkaline phosphatase; ALT, alanine transaminase; APRI, AST to platelet ratio index; AST, aspartate transaminase; CBC, complete blood count; CRP, C-reactive protein; FALD, Fontan-associated liver disease; GGT, gamma-glutamyl transpeptidase; INR, international normalized ratio; PTT, partial thromboplastin time; US, ultrasound; WBC, white blood cell.
